# Implementing AI in Hospitals to Achieve a Learning Health System: Systematic Review of Current Enablers and Barriers

**DOI:** 10.2196/49655

**Published:** 2024-08-02

**Authors:** Amir Kamel Rahimi, Oliver Pienaar, Moji Ghadimi, Oliver J Canfell, Jason D Pole, Sally Shrapnel, Anton H van der Vegt, Clair Sullivan

**Affiliations:** 1 Queensland Digital Health Centre Faculty of Medicine The University of Queensland Brisbane Australia; 2 Digital Health Cooperative Research Centre, Australian Government Sydney Australia; 3 The School of Mathematics and Physics The University of Queensland Brisbane Australia; 4 Business School The University of Queensland Brisbane Australia; 5 Department of Nutritional Sciences Faculty of Life Sciences and Medicine King’s College London London United Kingdom; 6 Dalla Lana School of Public Health The University of Toronto Toronto, ON Canada; 7 ICES Toronto, ON Canada; 8 Metro North Hospital and Health Service Department of Health Queensland Government Brisbane Australia

**Keywords:** life cycle, medical informatics, decision support system, clinical, electronic health records, artificial intelligence, machine learning, routinely collected health data

## Abstract

**Background:**

Efforts are underway to capitalize on the computational power of the data collected in electronic medical records (EMRs) to achieve a learning health system (LHS). Artificial intelligence (AI) in health care has promised to improve clinical outcomes, and many researchers are developing AI algorithms on retrospective data sets. Integrating these algorithms with real-time EMR data is rare. There is a poor understanding of the current enablers and barriers to empower this shift from data set–based use to real-time implementation of AI in health systems. Exploring these factors holds promise for uncovering actionable insights toward the successful integration of AI into clinical workflows.

**Objective:**

The first objective was to conduct a systematic literature review to identify the evidence of enablers and barriers regarding the real-world implementation of AI in hospital settings. The second objective was to map the identified enablers and barriers to a 3-horizon framework to enable the successful digital health transformation of hospitals to achieve an LHS.

**Methods:**

The PRISMA (Preferred Reporting Items for Systematic Reviews and Meta-Analyses) guidelines were adhered to. PubMed, Scopus, Web of Science, and IEEE Xplore were searched for studies published between January 2010 and January 2022. Articles with case studies and guidelines on the implementation of AI analytics in hospital settings using EMR data were included. We excluded studies conducted in primary and community care settings. Quality assessment of the identified papers was conducted using the Mixed Methods Appraisal Tool and ADAPTE frameworks. We coded evidence from the included studies that related to enablers of and barriers to AI implementation. The findings were mapped to the 3-horizon framework to provide a road map for hospitals to integrate AI analytics.

**Results:**

Of the 1247 studies screened, 26 (2.09%) met the inclusion criteria. In total, 65% (17/26) of the studies implemented AI analytics for enhancing the care of hospitalized patients, whereas the remaining 35% (9/26) provided implementation guidelines. Of the final 26 papers, the quality of 21 (81%) was assessed as poor. A total of 28 enablers was identified; 8 (29%) were new in this study. A total of 18 barriers was identified; 5 (28%) were newly found. Most of these newly identified factors were related to information and technology. Actionable recommendations for the implementation of AI toward achieving an LHS were provided by mapping the findings to a 3-horizon framework.

**Conclusions:**

Significant issues exist in implementing AI in health care. Shifting from validating data sets to working with live data is challenging. This review incorporated the identified enablers and barriers into a 3-horizon framework, offering actionable recommendations for implementing AI analytics to achieve an LHS. The findings of this study can assist hospitals in steering their strategic planning toward successful adoption of AI.

## Introduction

### Background

The growing adoption of electronic medical records (EMRs) in many high-income countries has resulted in improvements in health care delivery through the implementation of clinical decision support systems at the point of care [1]. To meet the ever-accelerating demands for clinical care, various innovative models have been developed to harness the potential of EMR data [2-4]. These new care models aim to enable health care organizations to achieve the quadruple aim of care, which includes enhancing patient experience, advancing providers’ experience, improving the health of the population, and reducing health care costs [5].

Artificial intelligence (AI) holds the potential to improve health system outcomes by enhancing clinical decision support systems [6,7]. AI aims to augment human intelligence through complicated and iterative pattern recognition, generally on large data sets that exceed human abilities [8]. While a large body of academic literature has demonstrated the efficacy of AI models in various health domains, most of these models remain as proof of concept and have never been implemented in real-world workflows [9]. This demonstrates the relatively inconsequential endeavors of many AI studies that fail to produce any meaningful impact in the real world. Even with the substantial investments made by the health industry, the implementation of AI analytics in complex clinical practice is still at an early stage [10]. In a limited number of instances, AI has been successfully implemented, largely for nonclinical uses such as service planning or trained on limited static data sets such as chest x-rays or retinal photography [11]. The factors influencing the success or failure of AI implementations in health are poorly investigated [12]. Understanding these barriers and enablers increases the likelihood of successful implementation of AI for the digital transformation of the health system [13,14], ultimately aiding in achieving the quadruple aim of health care [5].

### Toward the Digital Transformation of Health Care

**Figure 1:**
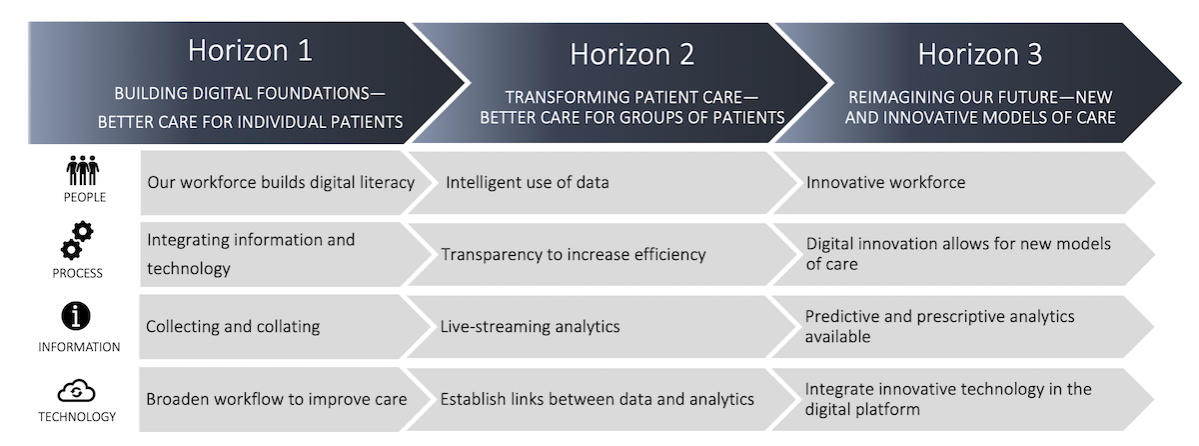
The 3-horizon framework for digital health transformation (adapted from Sullivan et al [15] with permission from CSIRO Publishing).

A 3-horizon framework has been previously published to help health systems create an iterative pathway for successful digital health transformation (Figure 1 [15]). Horizon 1 aims to optimize the routine collection of patient data during every interaction with the health system. In horizon 2, the data collected during routine care are leveraged in real or near real time to create analytics. Finally, in horizon 3, the insights from data and digital innovations are collated to develop new models of care. A health care system focused on continuous improvement is referred to as a learning health system (LHS) that uses routinely collected data to monitor and enhance health care outcomes consistently [16]. When health care organizations reach the third horizon, they can leverage data in near real time to create ongoing learning iterations and enhance patient care, leading to the establishment of an LHS [17].

Regarding the 3-horizon model, EMRs are the foundation of horizon 1 (Figure 1). While many health organizations have successfully adopted EMRs into their existing workflows, the transition to horizons 2 and 3 has been challenging for many of these health care facilities [18]. A critical phase in this transition involves moving beyond the capture of EMR data for delivering analytics, including AI, aiming to improve clinical outcomes. There is little published evidence to assist health systems in making this transition [19,20].

### Analysis of Prior Work

Before conducting our review, we performed a manual search on Google Scholar using our Medical Subject Heading (MeSH) terms along with the “review” keyword to identify previous review papers that aimed at reviewing studies on the implementation of clinical AI in health care settings. We also included review papers known to our research team. Between 2020 and 2022, we identified 4 reviews that were relevant to the implementation of AI in health care systems [21-24]. Overall, these papers reviewed 189 studies between 2010 and 2022. The characteristics of these reviews, outlined in Table 1, were the year of publication, the targeted care settings, the source of data, the predictive algorithm, and whether the predictive algorithm was implemented.

**Table 1:** The inclusion criteria for this study and previous work.

Study	Year	Health care setting	Data source	Predictive algorithm	Implementation state
Lee et al [22]	2020	Any	EMR^a^	Any	Implemented
Wolff et al [23]	2021	Any	Any	AI^b^ and ML^c^	Implemented
Sharma et al [21]	2022	Any	Any	AI and ML	Implemented
Chomutare et al [24]	2022	Any	Any	AI and ML	Implemented or developed
Our study	2023	Hospitals	EMR	AI and ML	Implemented or guidelines

^a^EMR: electronic medical record.

^b^AI: artificial intelligence.

^c^ML: machine learning.

The prior works identified 20 enablers and 13 barriers to AI implementation in health care across 4 categories: people, process, information, and technology (Multimedia Appendix 1 [21-24]). Overall, the findings derived from these review papers hold significant potential in providing valuable insights for health systems to navigate the path toward digital health transformation. One prevailing shortcoming of these studies is the absence of alignment with evidence-based digital health transformation principles to provide health care organizations with actionable recommendations to enable an LHS [17], therefore limiting their applicability for strategic planning within hospital organizations.

### Research Significance and Objectives

Hospitals are intricate hubs within the health care ecosystem, playing a central role in providing comprehensive medical care and acting as crucial pillars supporting the foundations of health care systems worldwide. Understanding the factors influencing the success or failure of AI in hospitals provides valuable insights to optimize the integration of these emerging technologies into hospital facilities. While the previous reviews included all health care settings [21-24], our study only focused on hospital settings. Given the limited instances regarding the implementation of AI in hospital facilities, this study explored the real-world case studies that have practically reported their AI implementation solutions in hospital facilities, aiming to synthesize the evidence of enablers and barriers within their implementation process. In addition to the inclusion of these implementation case studies, we incorporated implementation guidelines as they can potentially assist in the overall understanding of AI implementation in hospitals. This study also focused on aligning the evidence of enablers and barriers within the 3-horizon framework [15], offering a way to establish an empirical infrastructure. As a result, this can enable health care organizations to learn, adapt, and accelerate progress toward an LHS [25].

This review investigated the following research questions (RQs): (1) What enablers and barriers are identified for the successful implementation of AI with EMR data in hospitals? (RQ 1) and (2) How can the identified enablers of and barriers to AI implementation lead to actions that drive the digital transformation of hospitals? (RQ 2).

In addressing these questions, our objectives were to (1) conduct a systematic review of the literature to identify the evidence of enablers of and barriers to the real-world implementation of AI in hospital settings and (2) map the identified enablers and barriers to a 3-horizon framework to enable the successful digital health transformation of hospitals to achieve an LHS.

## Methods

### Search Strategy

This study followed an extended version of the PRISMA (Preferred Reporting Items for Systematic Reviews and Meta-Analyses) guidelines to outline the review methodology with comprehensive details [26]. PubMed, Scopus, Web of Science, and IEEE Xplore were searched on April 13, 2022. We reviewed prior work to determine potential MeSH keywords relevant to our study [21-24]. A research librarian helped with the definition of the MeSH keywords in PubMed and the translation of that search strategy to all platforms searched. The search strategies were applied across the 4 databases (Multimedia Appendix 2). The MeSH keywords used to search PubMed were as follows: *product lifecycle management*, *artificial intelligence*, *machine learning*, *deep learning*, *natural language processing*, *neural networks*, *computer*, *deep learning*, *big data*, *hospital*, *inpatient*, *medical*, *clinic*, *deploy*, *integrate*, *monitor*, *post prediction*, *data drift*, and *regulatory*. Using the Boolean operator *OR*, their synonyms were joined to form search phrases. Combining search phrases using the *AND* operator produced the final search string. We incorporated the term “data drift” to the title and abstract, and full-text search as it is a prominent concept for the continuous integration of AI. The term “regulatory” was also added to our search criteria because it is a relevant term for the implementation of AI in health care within the domain of software as a medical device. The reference lists of the included studies were examined to ensure that all relevant papers were included.

### Eligibility Criteria

The inclusion criteria were articles published from January 1, 2010, to April 13, 2022, that included case studies and guidelines on the implementation of AI analytic tools in hospital settings using EMR data. Given the scarcity of real-world AI tools in hospital settings, especially the scarcity of published case studies of unsuccessful implementations of clinical AI tools, we specifically included case studies that successfully implemented AI within hospitals to understand lessons learned and provide use cases that other jurisdictions may learn from. On the basis of a review of frameworks for AI implementation in health care practice, we defined the term *implementation* as “an intentional effort designed to change or adapt or uptake interventions into routines” [19]. The term “barrier” was defined as “experiences that impeded, slowed, or made implementations difficult in some way” [20]. In contrast, the term *enablers* was defined as factors, experiences, or processes that facilitated the implementation process. Studies conducted in community or primary care settings were excluded as our main focus was hospital facilities. Studies that did not use AI models were also excluded. We also eliminated non–English-language and conference articles. Studies that focused on regulatory domains and challenges, opportunities, requirements, and recommendations were also excluded as they did not demonstrate real-world AI implementation. The selection of studies was based on the criteria specified in Textbox 1.

**Inclusion criteria for this study.**

**Inclusion criteria**

- Population: adults (aged ≥18 y); inpatients
- Intervention: successfully implemented artificial intelligence (AI) and machine learning (ML) tools using hospital electronic medical record data
- Study design: case studies that implemented AI and ML in the real world; guidelines on the real-world implementation of AI and ML
- Publication date: January 2010 to April 2022
- Language: English

**Exclusion criteria**

- Population: nonadults (aged <18 y); outpatients
- Intervention: traditional statistical methods; rule-based systems; systems without AI and ML
- Study design: studies without implementation of AI and ML; studies focused on AI and ML development, regulatory-related domains, challenges, opportunities, and recommendations; conference papers; primary care or community settings
- Language: non-English

### Screening

For the screening and data extraction procedures, the Covidence (Veritas Health Innovation) systematic review software was used [27]. A 2-stage screening process was performed with the involvement of 2 reviewers (AKR and OP). In the initial stage, the reviewers assessed the relevance of titles and abstracts based on the inclusion criteria. Subsequently, in the second stage, the full texts of the included articles were reviewed by AKR and OP independently. Consensus was reached through discussion between the reviewers whenever necessary.

### Data Extraction and Synthesis

AKR and OP conducted the procedure of data extraction. The following study characteristics were extracted from all final included studies: country, clinical setting, study type (case study or guideline), and aim of study. With the adoption of EMR as a prerequisite for AI development, our focus was on extracting evidence of enablers and barriers solely within horizons 2 (implementation) and 3 (creating new models of care). In total, 2 reviewers (AKR and OP) independently extracted evidence regarding enablers and barriers (RQ 1), subsequently reaching consensus through weekly discussions and analysis. The extracted data were disseminated among our research team for review and to gather additional feedback.

To address the second RQ (RQ 2), we mapped the findings from previous reviews along with the found factors in this study across horizons 2 and 3 of the digital transformation framework [15]. Following the data extraction phase, 2 reviewers independently mapped the identified enablers and barriers to 4 categories (people, process, information, and technology). During the mapping of a given enabler or barrier, if it was related to the development of AI analytics, it was mapped to horizon 2 considering its relevance across the 4 domains (people, technology, information, and processes). When an enabler or barrier was associated with the postdevelopment phase focusing on establishing new care models, it was mapped to horizon 3. Consensus was reached between AKR and OP through a meeting to finalize the mapping phase.

### Quality Assessment

For the included use case studies, we used the Mixed Methods Appraisal Tool (MMAT) [28] to conduct a quality assessment. The choice of the MMAT was suitable as the included use case studies exhibited a range of qualitative, quantitative, and mixed methods designs. For evaluating the methodology of guideline studies, we followed the ADAPTE framework [29]. With 9 modules for guideline development, this framework was designed to streamline and enhance the process of creating guidelines within the health domain. The quality assessment was conducted independently by 2 authors (AKR and OP), and any discrepancies were resolved through a meeting.

## Results

### Study Selection

**Figure 2:**
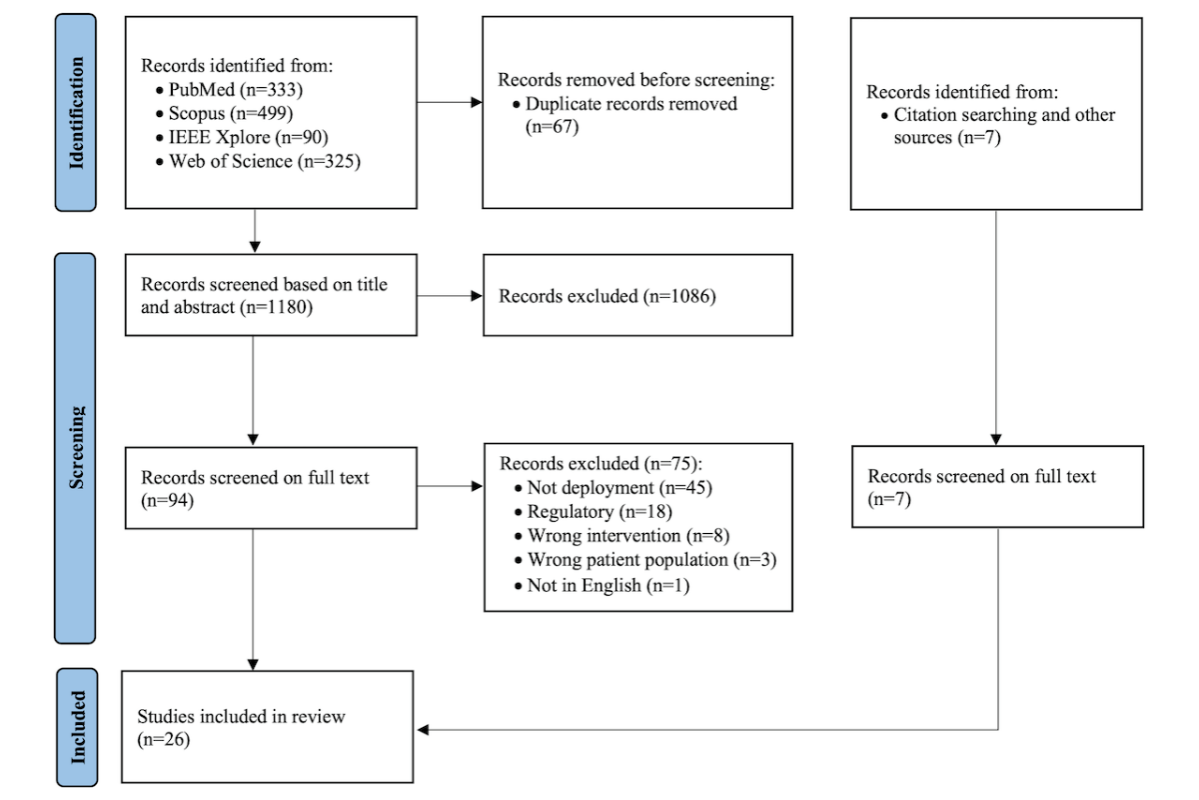
PRISMA (Preferred Reporting Items for Systematic Reviews and Meta-Analyses) flowchart for study selection.

The search strategy retrieved 1247 papers from PubMed, Scopus, IEEE Xplore, and Web of Science for analysis, and 67 (5.37%) duplicates were identified and eliminated using the EndNote (Clarivate Analytics) citation manager. After screening titles and abstracts, 92.03% (1086/1180) of the studies were removed as the inclusion criteria were not satisfied. A total of 7.97% (94/1180) of the papers remained for full-text review following title and abstract screening. In total, 48% (45/94) of papers were excluded because AI models were not implemented in clinical care. A total of 19% (18/94) of the studies were excluded because they focused on regulatory domains. In total, 9% (8/94) of the studies were excluded due to being the wrong intervention (eg, studies that did not develop AI models). A total of 3% (3/94) of the studies were found to have a clinical population that did not align with our inclusion criteria (eg, hospitalized patients). One study was not in English and was excluded. In addition, 7 studies were discovered by scanning the reference lists of the included articles. In total, 26 studies were included in this review, comprising 9 (35%) guideline studies and 17 (65%) papers with successful implementation examples (Table 2). Figure 2 presents the PRISMA flow diagram outlining the outcomes of this review.

**Table 2:** Characteristics of the studies included in this review.

Study, year	Country	Clinical setting	Study type	Aim of study	Enablers	Barriers
Wilson et al [30], 2021	United Kingdom	General	Guideline	To provide advice from health care experts on clinical AI^a^ development and implementation	A team of multidisciplinary experts, including clinicians, software developers, data scientists, and hospital IT staff Senior, experienced individuals can be particularly useful to overcome implementation barriers The appointment of a data champion Staff training in the data science field Using data scientists or a trusted research environment with appropriate tools can ensure adequate data privacy A common language with necessary terminologies is suggested within the CST^b^ Clinicians can assist in understanding and resolving the quality and reliability of AI solutions	HCPs’^c^ inexperience with AI The integration of disparate data sources is one of the barriers to AI solutions in the current workflow
Svedberg et al [31], 2022	Sweden	General	Guideline	To develop an AI implementation framework in health care To conduct AI implementation studies to provide direction for further improvement of the framework To implement the proposed framework in routine care	The co-design process among clinicians, data scientists, and end users The national and regional initiatives to facilitate AI implementation into practice Several major investments facilitated the establishment of the infrastructure design and development of this study Literature review and the existing theory-driven frameworks and strategies Technological knowledge and awareness of challenges, including social, cultural, and organizational barriers	Lack of accessibility of AI implementation science to individuals who could potentially benefit from it
Subbaswamy and Saria [32], 2019	United States	General	Guideline	To explain data shift and overview the types of existing solutions	Graphical representation can be used to assess the stability of AI models and identify potential performance shifts but requires domain for interpretation Proactive learning approaches allow models to be stable against anticipated shifts in the future, including the use of stable algorithms that are robust to future shift	Data set shift is prevalent and problematic in clinical AI settings and needs to be accounted for to prevent performance decay
Pianykh et al [33], 2020	United States	Radiology	Guideline	To examine the key principles and issues involved in integrating AI with continuous learning in radiology	Radiologists and clinicians are important to the successful implementation of continuous-learning AI to provide feedback Continuous learning is a viable method to combat data drift	Not specified
Leiner et al [34], 2021	The Netherlands	Radiology	Guideline	To demonstrate the necessity for a vendor-neutral AI implementation infrastructure To provide a plan for a vendor-neutral AI implementation infrastructure To discuss prominent issues, including governance, quality control, and ethics	A team of multidisciplinary experts, including clinicians, data scientists, and IT staff Platforms are suggested as vendor-neutral infrastructures shared by researchers and clinicians and allow AI systems to receive iterative feedback from clinicians The accessibility of the AI results at the time of care without requiring physicians to switch workstations or launch specialized software Consistency between AI implementation methods used within one hospital The messaging standards, such as HL7^d^ Using the containerization concept to concurrently run multiple instances of AI analytics Training end users and clinicians for using and interpreting AI results	Not specified
Gruendner et al [35], 2019	Germany	General	Guideline	To implement a secure platform to develop and deploy ML^e^ models in health care settings	The FHIR^f^ standard was used to exchange health data between different health care points in a consistent manner The OMOP-CDM^g^ database structure was used as a standard method to organize health care data consistently across various data points. This also enabled the availability of data to researchers and end users. Containerization allowed for a flexible development environment. It enabled clinicians and ML developers to collaborate and improve performance. The proposed platform provides scientists with a secure, privacy-preserving, flexible research infrastructure to develop and deploy statistical models within a hospital’s IT infrastructure Using appropriate data privacy techniques can allow for model training using data from multiple hospitals in parallel Collaboration among the research team	Predictions can be extremely slow with large input data due to hardware limitations; therefore, the AI may output results not in real time Generalizable platforms such as KETOS are versatile, but as a result, they are relatively inefficient and may require further customizations and fine-tunings at the local level
Eche et al [36], 2021	United States	Radiology	Guideline	To provide strategies to tackle overfitting and underspecification of AI models	Underspecification (the lack of generalizability) can be addressed with the use of artificial or real shifts in test data	Overfitting and underspecification can negatively impact the generalizability of AI in health care There is a trade-off between performance and generalizability when addressing underspecification
Allen et al [37], 2021	United States	Radiology	Guideline	Guideline of evaluation of AI in a radiology setting before implementation in the workflow to assist in purchase decisions and monitoring of the performance afterward	Enriched site-specific data can facilitate AI evaluation, allowing that the target population is well-represented before implementation In the AI evaluation process, capturing the metadata about equipment manufacturers, the protocol used, and demographics in the AI data registry can reveal performance decline and show whether the decline is related to specific machines or manufacturers QA^h^ allows AI to perform according to the implementation requirements	Model evaluation can be difficult and restricted to larger, informatics-familiar institutions
Verma et al [38], 2021	Canada	General	Guideline	To provide an approach for developing and implementing AI in health care	Multidisciplinary team Safety monitoring Data quality User-friendly user interface Nondisruptive to the current workflow End-user trust Continuous evaluation of performance	Not reported
Wiggins et al [39], 2021	United States	Radiology	Case study	To develop an AI solution that can generate, consume, and provide outcomes within the clinical radiology process	Collaboration among developers, radiologists, and AI vendors Interoperability standards and robust methodologies, such as HL7, FHIR, and SOLE^i^ The use of metadata such as hardware or software specifications Radiologists should be able to provide feedback on AI results Raising awareness and providing the required training regarding the potential of AI technologies among clinicians and patients can help increase AI adoption	Not reported
Wang et al [40], 2021	China	Radiology	Case study	To create an AI system that analyzes CT^j^ scans automatically to promptly detect COVID-19 pneumonia in hospitals	Co-designing with clinicians The AI model was externally validated to assess the generalizability before deployment Preconfigured model development allowed for very quick deployment Continuously collected data can lead to better generalizability of AI products and are considered a crucial aspect of epidemic response	Lack of reception of continuous data for retraining the model may result in data drift and underfitting
Strohm et al [41], 2020	The Netherlands	Radiology	Case study	To explore barriers to and enablers of AI implementation in radiology	Collaboration among HCPs in radiology Financial challenges in the Dutch health care system The optimism toward AI potential The existing strategies and initiatives in digital health The appointment of a data champion	Inconsistent efficacy of AI output Lack of robust implementation procedures Unclear added value of AI applications in routine care Trust issue of HCPs
Soltan et al [42], 2022	United Kingdom	ED^k^ triage	Case study	To implement an AI application to screen patients with COVID-19 in an ED and perform multicenter external validation	Conducted multicenter validation across 4 hospitals, including both temporal and geographical validations Deployment occurred in parallel with the preexisting method, allowing for a direct comparison of performance The AI only using laboratory tests already routinely done allowed for minimal interruption of regular clinical workflow Temporal and geographical external validation allowed for the assessment of the generalizability of the AI tool	Validation only performed in 1 geographical region
Sohn et al [43], 2020	United States	Radiology	Case study	To develop an infrastructure for the implementation of ML models in routine radiology workflow	Collaboration of a multidisciplinary team The minimum disruption to the current workflow can increase the AI uptake An open-source pipeline facilitates the integration of additional algorithms An ML model agnostic to the hospital systems for easier modification and retraining without impacting the existing infrastructure The use of a QA framework by end users, clinicians, and software testers to identify model errors and submit those errors for model update Minimum disruption to the existing radiology workflow QA evaluation A dedicated server for the AI applications	Preexisting pipelines for clinical AI deployment often rely on third-party software, which can be problematic due to complexity, privacy, and maintenance issues
Pierce et al [44], 2021	United States	Radiology	Case study	To implement an AI-enabled mobile x-ray scanner detecting pneumothoraxes in a radiology clinical workflow	Compatibility of the clinic’s system with the vendor along with the vendor’s willingness to collaborate Granting user access privileges according to their specific roles Staff training The model received continuous training Training and education of users in the use of AI can be beneficial Minimum disruption to the current workflow	Not reported
Kanakaraj et al [45], 2022	United States	Radiology	Case study	To develop and demonstrate a clinical image AI validation tool with a convenient user-friendly front end while meeting important security and privacy standards	Use of secure software—PACS^l^ image management, HTTPS service, and REDCap^m^ database The AI imaging incubator successfully provided an architecture for executing clinical AI models and displaying results in a clinician-friendly manner while meeting key security and privacy standards (HIPAA^n^ compliance)	Lack of appropriate procedure to capture users’ feedback for continuous improvement of AI model
Jauk et al [46], 2020	Austria	General	Case study	To implement ML models to forecast the occurrence of delirium among patients admitted to hospitals	Clinical staff were involved in the implementation process Training for nurses and physicians involved is beneficial	Performance analysis can be complicated for early-warning intervention AI systems The incidence of delirium was lower than anticipated, impacting the calibration Sometimes, the algorithm would underperform on patients with fewer previous hospital stays due to reduced EHR^o^ data
Davis et al [47], 2019	United States	General	Case study	To outline a procedure for selecting updating methods to combat clinical prediction model drift	The procedure effectively recommended updating methods proportional to the need This procedure can be applied to any type of model The procedure is conservative compared with others	The procedure provides no guarantee of clinically appropriate improvement to model performance
Blezek et al [48], 2021	United States	Radiology	Case study	To outline and demonstrate a system for general AI deployment in radiology and discuss use cases and requirements	The Agile development approach was used to deliver the AI product Radiology IT support was significantly involved Computational and storage resources were appropriately configured to properly handle the current and future processing requirements Radiologists received training on the use of the new system Custom solutions can fit and function seamlessly in clinical workflows but are susceptible to some issues Radiologists approved of the ability to conveniently decide the correctness of the results and the system’s seamless and intuitive integration into their workflow	Vended implementation platforms are also imperfect
Pantanowitz et al [49], 2020	United States	Pathology	Case study	To clinically validate an AI algorithm for detecting prostate adenocarcinoma, grade tumors, and detect clinically important features To deploy the AI algorithm in clinical workflow	Substantial increase in pathology workload and job complexity makes it a prime candidate for AI uptake External validation The use of unseen data sets for performance validation Small calibration data set was effective for adapting the algorithm to a new environment Combining target categories into clinically significant groups reduced computational requirements, allowing for real-time analysis	Discrepancy in labeling data due to discordance among physicians for cancer grading
Fujimori et al [50], 2022	Japan	ED	Case study	To evaluate the enablers of and barriers of implementing AI in emergency care	Data explanation and visualization were used to justify the alerts Robust validations are required to avoid undesired consequences Alert fatigue was avoided by processing background information and presenting visual data Training clinicians	Low performance in workflow Alert fatigue The risk of bias on a clinician’s decision when using the AI application
Joshi et al [20], 2022	United States	General	Case study	To examine the implementation of a sepsis CDS^p^ tool with ML models and rule-based approach from the viewpoint of those leading the implementation	Ease of integration and ability to customize the AI model	Difficulties with the definition of optimal alerts Alerts were said to be disruptive to the workflow Alert fatigue Concerns about the clinical relevance of the new system Difficult to explain and understand ML outputs Trust issue with the output due to misunderstanding the output High financial cost
Pou-Prom et al [51], 2022	Canada	General	Case study	To develop an AI application for predicting the risk of clinical deterioration in hospitals	Multidisciplinary team Security measures were adopted Clinical relevance to the targeted cohort Temporal validation Conducted a pilot test to understand the model output User training Model update to avoid data drift	Lack of external validation Lack of generalizability
Baxter et al [52], 2020	United States	General	Case study	To identify barriers to AI uptake in workflow	Co-design with end users	End users’ concerns about whether the new solutions are relevant to their workflow Potential disruption to the routine workflow and unintended consequences Lack of customization capability
Sandhu et al [53], 2020	United States	ED	Case study	To examine the variables influencing the implementation of ML applications for predicting sepsis incidence	Co-design with nurses and clinical staff Introduced a new job title responsible for the integration Having the required clinical knowledge about sepsis Training end users	Clinicians’ trust Lack of understanding of the output Alert fatigue Disruption to the workflow
Sendak et al [54], 2020	United States	ED	Case study	To report a deep learning sepsis detection and management system	Multidisciplinary team Co-design with clinical staff Hospital leaders and external research partners Training staff Data scientists with the required clinical background Personnel time for integration of new ML system Shared infrastructure for development and deployment	Lack of evidence-based implementation guidelines Disruption to the workflow Lack of feedback loop for continuous updating

^a^AI: artificial intelligence.

^b^CST: collaborative science team.

^c^HCP: health care provider.

^d^HL7: Health Level 7.

^e^ML: machine learning.

^f^FHIR: Fast Healthcare Interoperability Resources.

^g^OMOP-CDM: Observational Medical Outcomes Partnership Common Data Model.

^h^QA: quality assurance.

^i^SOLE: Standardized Operational Log of Events.

^j^CT: computerized tomography.

^k^ED: emergency department.

^l^PACS: picture archiving and communication system.

^m^REDCap: Research Electronic Data Capture.

^n^HIPAA: Health Insurance Portability and Accountability Act.

^o^EHR: electronic health record.

^p^CDS: clinical decision support.

### Study Characteristics

Table 2 outlines the characteristics of the included studies in this review. The publication dates of the included studies ranged from 2019 to 2022 [20,30-54]. In total, 65% (17/26) of the studies were case studies on the implementation of AI in hospitals [20,39-54], whereas the remaining 35% (9/26) were implementation guidelines [30-38].

Of the 26 identified studies, 15 (58%) originated from the United States [20,32,33,36,37,39,43-45,47-49,52-54]; 2 (8%) originated from the United Kingdom [30,42]; 2 (8%) originated from the Netherlands [34,41]; and 1 (4%) originated from China [55], Australia [46], Japan [50], Canada [51], Austria [46], Germany [35], and Sweden [31] each.

Radiology was the clinical setting in 46% (12/26) of the studies [33,34,36,37,39-41,43-45,48,49]. A total of 38% (10/26) of the studies were conducted in general inpatient wards [20,30-32,35,38,46,47,51,52], and 15% (4/26) were conducted in emergency departments [42,50,53,54].

### Quality Assessment

Regarding the 35% (9/26) of guideline studies, none fully adhered to the ADAPTE framework [29]. Although these included guideline studies had clear scopes and purposes aligned with this review, they all lacked details concerning the assessment of quality, external validation, and aftercare planning procedures. The details of this assessment for all the guideline studies can be found in Multimedia Appendix 3 [20,30-54].

With respect to the 65% (17/26) of case studies, they were classified into 3 groups: quantitative descriptive (12/17, 71%) [39,40,42-49,51,54], qualitative (4/17, 24%) [20,41,52,53], and mixed methods (1/17, 6%) [50]. Overall, 5 of the case studies met the MMAT criteria: all 4 (80%) qualitative studies and the one mixed methods study. The remaining 71% (12/17) of quantitative descriptive studies failed to fully adhere to the MMAT criteria. In all but 17% (2/12) of these quantitative descriptive studies, an appropriate data sampling strategy was not used to represent their target population [40,49]. The statistical analysis of the findings was assessed as appropriate in 58% (7/12) of the quantitative descriptive studies [42,43,46,47,49,51,54]. Overall, our assessment revealed that the quality of 81% (21/26) of the included studies was poor due to insufficient reporting of their methodologies (Multimedia Appendix 3).

### RQ Findings

#### RQ 1A Findings: Enablers of AI Implementation in Hospitals

A total of 28 enablers extracted from both prior work and this study (n=8, 29% were new enablers identified in our study) are presented in Table 3. Most of these newly identified enablers (7/8, 88%) related to the information and technology categories, highlighting the potential opportunities for hospitals regarding data readiness and required technologies for the successful implementation of AI. A total of 54% (15/28) of the enablers were shared findings between the previous reviews and this study.

**Table 3:** Consolidated view of research question 1A (enablers to artificial intelligence [AI] implementation; N=26)^a^.

Horizon and category			Source		Studies, n (%)	
			Previous studies	This study		
**Horizon 2: creating AI analytics**						
	**People**					12 (46)
		Enabler 1: multidisciplinary team	Sharma et al [21]	Blezek et al [48] Pierce et al [44] Pou-Prom et al [51] Sendak et al [54] Sohn et al [43] Strohm et al [41] Wang et al [40] Wiggins et al [39] Gruendner et al [35] Leiner et al [34] Verma et al [38] Wilson et al [30]		
		Enabler 2: experienced data scientists	—^b^	Sendak et al [54] Strohm et al [41] Wilson et al [30]		
	**Process**					22 (85)
		Enabler 3: co-design with clinicians	Sharma et al [21]	Baxter et al [52] Pierce et al [44] Sandhu et al [53] Sendak et al [54] Sohn et al [43] Strohm et al [41] Wang et al [40] Wiggins et al [39] Gruendner et al [35] Pianykh et al [33] Svedberg et al [31] Wilson et al [30]		
		Enabler 4: robust performance monitoring and evaluation	Sharma et al [21] Chomutare et al [24]	Blezek et al [48] Fujimori et al [50] Pou-Prom et al [51] Sohn et al [43] Soltan et al [42] Allen et al [37] Verma et al [38]		
		Enabler 5: seamless integration	Sharma et al [21] Lee et al [22]	Blezek et al [48] Pierce et al [44] Sohn et al [43] Soltan et al [42] Leiner et al [34] Verma et al [38]		
		Enabler 6: organizational resources	Sharma et al [21] Lee et al [22] Chomutare et al [24]	Strohm et al [41] Wiggins et al [39] Svedberg et al [31] Wilson et al [30]		
		Enabler 7: evidence of clinical and economic AI added value	Sharma et al [21] Wolff et al [23] Chomutare et al [24]	Joshi et al [20] Blezek et al [48] Strohm et al [41]		
		Enabler 8: addressing data shift	Lee et al [22]	Wang et al [40] Davis et al [47] Eche et al [36]		
		Enabler 9: improved team communication	Sharma et al [21]	—		
	**Information**					9 (35)
		Enabler 10: data quality	Chomutare et al [24]	Pantanowitz et al [49] Pou-Prom et al [51] Wang et al [40] Wiggins et al [39] Allen et al [37]		
		Enabler 11: data security	Lee et al [22]	Kanakaraj et al [45] Pou-Prom et al [51] Gruendner et al [35]		
		Enabler 12: data visualization	—	Fujimori et al [50] Subbaswamy and Saria [32]		
	**Technology**					15 (58)
		Enabler 13: continuous learning capability	Sharma et al [21]	Pierce et al [44] Pou-Prom et al [51] Wang et al [40] Pianykh et al [33] Subbaswamy and Saria [32]		
		Enabler 14: containerization	—	Pierce et al [44] Sohn et al [43] Wang et al [40] Gruendner et al [35] Leiner et al [34]		
		Enabler 15: interoperability	Chomutare et al [24]	Kanakaraj et al [45] Wiggins et al [39] Gruendner et al [35] Leiner et al [34]		
		Enabler 16: shared infrastructure	—	Blezek et al [48] Sendak et al [54] Gruendner et al [35] Leiner et al [34]		
		Enabler 17: customization capability	Sharma et al [21] Lee et al [22] Wolff et al [23]	Joshi et al [20] Blezek et al [48] Sohn et al [43]		
		Enabler 18: vendor-agnostic infrastructure	—	Sohn et al [43] Leiner et al [34]		
		Enabler 19: computational and storage resources	—	Blezek et al [48] Pantanowitz et al [49]		
		Enabler 20: alert considerations	—	Fujimori et al [50]		
		Enabler 21: ease of integration	—	Joshi et al [20]		
**Horizon 3: implementation of new models of care**						
	**People**					8 (31)
		Enabler 22: skilled end users	Sharma et al [21] Lee et al [22] Chomutare et al [24]	Blezek et al [48] Jauk et al [46] Pierce et al [44] Sandhu et al [53] Sendak et al [54] Gruendner et al [35] Pianykh et al [33] Wilson et al [30]		
		Enabler 23: hospital leadership	Chomutare et al [24]	Sendak et al [54]		
		Enabler 24: innovation champions	Sharma et al [21] Lee et al [22] Chomutare et al [24]	—		
	**Process**					9 (35)
		Enabler 25: staff training	Chomutare et al [24]	Blezek et al [48] Fujimori et al [50] Jauk et al [46] Pierce et al [44] Pou-Prom et al [51] Sandhu et al [53] Sendak et al [54] Leiner et al [34] Wilson et al [30]		
		Enabler 26: provide incentives when using AI	Sharma et al [21] Lee et al [22]	—		
		Enabler 27: limiting non-AI solutions	Wolff et al [23]	—		
	**Information**					1 (4)
		Enabler 28: usability	Chomutare et al [24]	Verma et al [38]		

^a^Enablers identified in previous reviews and this review were mapped to 4 categories of the 3-horizon framework [15].

^b^Not specified.

Within the scope of the 3-horizon framework [15], most included studies in this paper (22/26, 85%) indicated that the process domain facilitated the development of AI analytics within horizon 2 [20,30,31,33-44,47,48,50-54]. Co-design with clinicians was the most commonly reported enabler in 46% (12/26) of the papers in horizon 2 [30,31,33,35,39-41,43,44,52-54]. The process domain was also highlighted as having a facilitative role in the creation of new care models with AI (horizon 3) in 35% (9/26) of the papers [30,34,44,46,48,50,51,53,54]. Training end users to adopt AI solutions and interpret the insights was reported in all these 9 studies as an enabling factor in horizon 3.

Technological factors were highlighted in 58% (15/26) of the studies as enablers within horizon 2 [20,32-35,39,40,43-45,48-51,54], with the most commonly reported factor being continuous learning capability of AI analytics [32,33,40,44,51] and containerization capability by providing separated development environments [34,35,40,43,44] and applying the interoperability techniques ensuring seamless integration of diverse formats of clinical data from different hardware and software sources [34,35,39,45].

Of all the included studies, 46% (12/26) [30,34,35,38-41,43,44,48,51,54] and 31% (8/26) [30,33,35,44,46,48,53,54] identified people-related enablers across horizons 2 and 3, respectively, with multidisciplinary teams in horizon 2 and trained end users in horizon 3 being the 2 most reported enablers.

Enabling factors related to the information domain were discussed in 35% (9/26) of the included studies in this review [32,35,37,39,40,45,49-51], with data quality being the most reported enabler of the successful implementation of AI in hospitals in >50% of these papers (5/9, 56%) [37,39,40,49,51]. The enablers of the AI adoption in hospitals were reported to include factors such as considerations of data security [35,45,51] and data visualization [32,50] in horizon 2 along with AI usability [38] solutions in horizon 3.

#### RQ 1B Findings: Barriers to AI Implementation in Hospitals

Overall, a total of 18 barriers to AI implementation in hospitals were extracted from both prior work and this study, with 5 (28%) found to be new in this study (Table 4). Most of these newly identified barriers (4/5, 80%) were related to the information and technology categories. A total of 50% (9/18) of the identified barriers were found to be shared findings between the previous work and this study. In our analysis, some factors played dual roles, acting as both enablers and barriers. For instance, “Seamless integration” served as an enabler (enabler 5; Table 3), whereas “Disruptive integration” acted as a barrier (barrier 3; Table 4). We reported both enablers and barriers with such reversed meanings to highlight the real-world complexities due to which such factors can exhibit this duality.

Regarding the 3-horizon framework [15], 58% (15/26) of the included studies in this review showed that the process domain hindered the development of AI within horizon 2 [20,31,37,40-43,45-47,50-54]. The lack of sufficient performance assessment within horizon 2 was the most commonly reported barrier in 27% (7/26) of the papers [37,41,42,46,47,50]. The factors related to the process domain were also reported as barriers to the implementation of AI within horizon 3, with 8% (2/26) of the papers reporting alert fatigue as an obstacle to AI adoption for creating new models of care [20,53].

Information-related factors were highlighted in 31% (8/26) of the studies as barriers within horizon 2 [20,35,36,46,51], with the most commonly mentioned one being poor data quality [20,35,36,46,51]. The challenge with data shift was reported as part of the information domain within horizon 3 [32].

Technology-related challenges in horizon 2 were identified in 19% (5/26) of the studies, including issues such as the lack of customization capability and computational limitations of hardware [35,43,48,50,52].

Within horizon 3, a total of 19% (5/26) of the included papers highlighted the barriers related to the people domain [20,30,41,50,53], with lack of trust by clinicians and inexperienced end users in using AI within their routine workflows being 2 barriers reported in these studies.

**Table 4:** Consolidated view of research question 1B (barriers to artificial intelligence [AI] implementation)^a^.

Horizon and category				Source					Studies, n (%)
				Previous studies		This study			
**Horizon 2: creating AI analytics**									
	**Process**								15 (58)
		Barrier 1: insufficient performance assessment	Chomutare et al [24]		Fujimori et al [50] Jauk et al [46] Soltan et al [42] Strohm et al [41] Davis et al [47] Allen et al [37]				
		Barrier 2: lack of standardized guidelines for AI implementation	Sharma et al [21] Wolff et al [23] Chomutare et al [24]		Pou-Prom et al [51] Sendak et al [54] Soltan et al [42] Strohm et al [41] Svedberg et al [31]				
		Barrier 3: disruptive integration	Lee et al [22] Chomutare et al [24]		Joshi et al [20] Baxter et al [52] Sandhu et al [53] Sendak et al [54]				
		Barrier 4: inadequate continuous learning	Chomutare et al [24]		Kanakaraj et al [45] Sendak et al [54] Wang et al [40]				
		Barrier 5: complexity of maintenance	Wolff et al [23] Chomutare et al [24]		Sohn et al [43]				
		Barrier 6: lack of clear consensus on alert definitions	—^b^		Joshi et al [20]				
		Barrier 7: insufficient data preprocessing	Wolff et al [23]		—				
	**Information**								8 (31)
		Barrier 8: poor data quality	Lee et al [22] Wolff et al [23] Chomutare et al [24]		Joshi et al [20] Jauk et al [46] Pou-Prom et al [51] Eche et al [36] Gruendner et al [35]				
		Barrier 9: data heterogeneity	—		Pantanowitz et al [49] Wilson et al [30]				
		Barrier 10: data privacy	—		Sohn et al [43]				
		Barrier 11: challenges with data availability	Lee et al [22] Wolff et al [23] Chomutare et al [24]		—				
	**Technology**								5 (19)
		Barrier 12: lack of customization capability	—		Baxter et al [52] Blezek et al [48] Sohn et al [43]				
		Barrier 13: computational limitations of hardware	—		Fujimori et al [50] Gruendner et al [35]				
**Horizon 3: implementation of new models of care**									
	**People**								5 (19)
		Barrier 14: inexperienced end users with AI output	Chomutare et al [24]		Joshi et al [20] Sandhu et al [53] Wilson et al [30]				
		Barrier 15: lack of clinician trust	Lee et al [22] Chomutare et al [24]		Fujimori et al [50] Sandhu et al [53] Strohm et al [41]				
	**Process**								2 (8)
		Barrier 16: alert fatigue	Lee et al [22] Chomutare et al [24]		Joshi et al [20] Sandhu et al [53]				
		Barrier 17: difficulties with understanding AI outputs	Chomutare et al [24]		—				
	**Information**								1 (4)
		Barrier 18: data shift	Lee et al [22]		Subbaswamy and Saria [32]				

^a^Barriers identified in previous reviews and this review were mapped to 4 categories of the 3-horizon framework [15].

^b^Not specified.

#### RQ 2 Findings: Mapping the Findings to the 3-Horizon Framework

**Figure 3:**
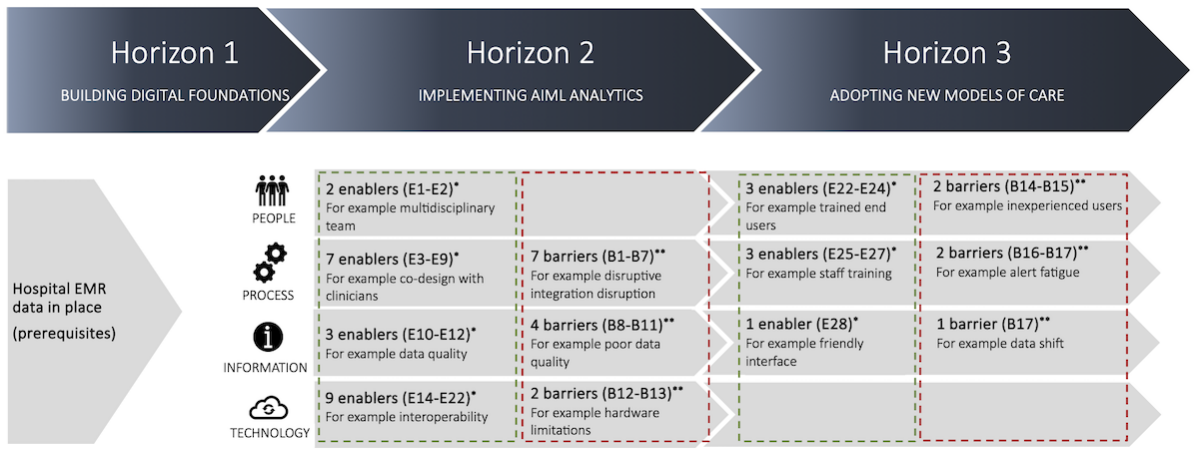
Mapping the identified enablers and barriers to the 3-horizon framework (adapted from Sullivan et al [15] with permission from CSIRO Publishing). *Enablers described in Table 3; **Barriers described in Table 4. AIML: artificial intelligence machine learning; B: barrier; E: enabler; EMR: electronic medical record.

The identified enablers and barriers to AI implementation in hospitals (RQ 1) were mapped to the 3-horizon framework [15] across 4 categories: people, process, information, and technology within horizons 2 and 3 (Figure 3 [15]).

In horizon 2, we identified a total of 21 enablers, with most associated with technology (n=9, 43%) and processes (n=7, 33%). Moving to horizon 3, a total of 7 enablers were identified, spanning the categories of people (n=3, 43%), processes (n=3, 43%), and information (n=1, 14%). Regarding barriers, horizon 2 presented a total of 13 barriers, with >50% (n=7, 54%) falling into the process category. In horizon 3, we identified a total of 5 barriers primarily distributed among the people (n=2, 40%), process (n=2, 40%), and information (n=1, 20%) categories.

## Discussion

### Principal Findings

The health care industry needs to adopt new models of care to respond to the ever-growing demand for health services. Over the last decade, the academic community has shown considerable interest in the application of AI to explore new innovative models of care. Despite the numerous papers published each year exploring the potential of AI in various health domains, only a few studies have been implemented into routine workflows. Investigating the factors that lead to the success or failure of AI in health care could potentially provide actionable insights for the effective implementation of AI in clinical workflows. In this review, we explored the current state of the literature focusing on the implementation of AI in hospitals. Our review of 26 studies revealed several enablers of and barriers to the implementation of AI in digital hospitals. Although our search for studies dated back to 2010, all 26 case studies and guidelines found in our study were published from 2019 onward. This is not surprising considering the significant progress made in AI implementation across many fields in recent years. Given such substantial advancements, implementation science needs to be further developed to accommodate these new AI innovations in health care [19]. This paper can serve as a road map for decision makers, presenting key actionable items to translate AI into hospital settings and leveraging it for potential new models of care.

While this paper extends the findings of previous reviews by examining the factors associated with AI implementation in health care [22-24], a significant aspect found in both previous reviews and our study underscores the significance of process-related factors for creating AI analytics. A large number of papers identified in this study (22/26, 85%) reported process factors as enablers of their AI implementation, aligning with the factors found in all previous reviews (enablers 3-9; Table 3). This commonality indicates the significant opportunity for hospitals to leverage their existing workflows as a strategic approach to enable AI adoption. In the context of developing innovative care models through AI analytics, obstacles associated with people (barriers 14 and 15; Table 4) were identified in 19% (5/26) of the included studies, consistent with findings in 2 previous reviews [22,24]. This highlights the influence of human factors in facilitating the integration of AI in practice.

Apart from the common findings between this and previous reviews, there are several novel aspects to this study. First, it centered specifically on hospitals, the largest and richest source of clinical data. Second, it incorporated AI implementation guidelines from the included studies, allowing for a broader understanding of AI implementation. Third, our review identified new enablers of AI implementation regarding technology and information that can facilitate AI implementation, including quality of data, shared infrastructure for continuous development, and capabilities regarding hardware resources. Fourth, this paper identified new barriers to AI implementation, with most of them being within the domains of process, information, and technology. These barriers included challenges such as data privacy, dealing with heterogeneous data, limitations with the customization of AI analytics, and ambiguity surrounding the design of alert definitions. Finally, the study findings were mapped to a 3-horizon framework encompassing 4 key categories: people, information, process, and technology. This framework offers a clear and practical road map for health care organizations planning to create new AI analytics.

It is important to note that, while our primary focus was on hospital facilities, the findings of this review may exhibit variations across other health care settings. For example, the incorporation of AI in outpatient care may demand different technological infrastructures to enable AI development. Future research can expand upon this study by investigating the evidence of enablers and barriers associated with AI implementation in wider health care settings, including primary care and outpatient care, as we expect that the outcomes of this study may differ in other health care settings. Moreover, the incorporation of studies related to regulatory aspects can be a crucial component for a more comprehensive understanding of the trajectory of AI adoption within health care systems.

### Toward AI Implementation in Hospitals

#### Actionable Recommendations

In this section, we consolidate the findings of this study and prior work within the scope of a 3-horizon framework [15] and provide recommendations for health care organizations that plan to implement AI analytics in hospitals (Textbox 2). These recommendations are not the ultimate solution but rather a flexible action plan to facilitate AI implementation and mitigate potential challenges regarding the digital transformation of hospitals.

**Recommendations for artificial intelligence (AI) adoption in hospitals.**

**Horizon 1: establishing digital infrastructure**

- Implement functional electronic medical record system
- Focus on improving data quality
- Maintain data privacy and security
- Facilitate data availability

**Horizon 2: create AI analytics**

- Co-design with multidisciplinary team
- Employ experienced data scientists
- Adopt interoperability methods
- Focus on AI usability
- Continuously develop and evaluate AI results
- Enhance data security and privacy
- Improve computational capabilities
- Focus on seamless integration
- Enhance customization capability
- Demonstrate AI added value
- Improve team communication
- Define design standards for AI output
- Focus on vendor-agnostic architecture

**Horizon 3: create new models of care**

- Restructure the clinical care models using insights from AI analytics
- Provide user training
- Continuously improve quality to produce reliable AI output and minimize data shift and alert fatigue
- Leverage hospital leaders to drive AI adoption
- Appoint innovation managers
- Provide incentive for using AI

#### Horizon 1: Establishing Digital Infrastructure

Data form the core of AI development to create clinical analytics. Some information barriers emerging in horizon 2, presented in Table 4, may be associated with challenges regarding EMR data, for example, quality of data (barrier 8), data heterogeneity (barrier 9), and data privacy (barrier 10). In the integration of EMR systems within hospital settings, careful attention must be paid to the functionality of the system to enable routine data collection to support the continuous development of AI analytics. Prioritizing the enhancement of data quality through the implementation of rigorous validation processes is a key factor in producing generalizable, reliable, and effective AI outputs. It is also imperative to ensure strict adherence to data privacy protocols during the EMR implementation, safeguarding sensitive patient information and maintaining ethical standards in handling health care data.

#### Horizon 2: Creating Analytics

Horizon 2 primarily focuses on data extraction and developing AI analytics. The successful implementation of AI in this horizon will be discussed within the following themes.

##### Form a Diverse Team of Experts

There is evidence suggesting that building a multidisciplinary team consisting of clinicians, nurses, end users, and data scientists can facilitate the successful design and implementation of AI in hospitals (enabler 1; Table 3). Experienced data scientists can potentially increase the success of AI in health care by ensuring accurate, reliable, and fair AI output in addition to identifying biases, handling complex medical data effectively, and optimizing AI algorithms (enabler 2; Table 3).

##### Enhance the Existing Processes

While horizon 2 revolves around technical aspects of AI implementation, the evidence indicates that involving clinicians, end users, and technical staff in the design and implementation stages is needed for successful integration (enabler 3; Table 3). The co-design strategy can alleviate challenges such as the lack of consensus on alert definitions (barrier 6; Table 4), leading to usability improvement (enabler 28; Table 3). Enhancing the understanding of AI output through training end users has the potential to alleviate concerns about the usability of AI output, fostering a smoother adoption of AI technologies in hospitals.

The studies recognized that minimizing workflow disruption is key for the successful implementation of AI (enabler 5; Table 3). To minimize workflow disruption and ensure a smooth transition when implementing new AI solutions in hospitals, it is important to engage end users from the early stage of the development process [56], although training and education should be provided to help staff members effectively incorporate the AI solution into their daily routines. For successful implementation of less disruptive technologies such as AI, it is recommended to establish a clear vision and communication by the leadership team (enabler 23; Table 3), have innovation champions (enabler 24; Table 3), and provide incentives (enabler 26; Table 3) to drive long-term adoption and habit formation [57].

Continuous AI development with the use of routinely collected data and clinicians’ feedback ensures that AI results accurately reflect the current clinical situations in hospital settings (enabler 13; Table 3). This can support clinicians in making more accurate diagnoses and treatment decisions by leveraging the latest insights derived from AI analytics. While insufficient assessment of AI performance in hospital settings is considered a prominent obstacle to successful implementation (barrier 1; Table 4), continuous development and monitoring helps avoid “data drift,” a phenomenon in which AI models lose accuracy over time due to changes in the data or environment [32,47].

##### Strive for Better Data Quality and Security

The studies indicated that the implementation of AI is hindered by data privacy concerns (barrier 10; Table 4). Hospitals can mitigate the risks associated with data handling and storage by adopting standardized data frameworks and interoperability techniques (enabler 15; Table 3). These measures help minimize vulnerabilities and enhance overall data security.

The quality of data in developing AI analytics refers to the accuracy, completeness, consistency, reliability, and relevance of the data used to implement AI analytics and is considered a crucial enabler for successful AI implementation in hospitals [58]. Hospitals are encouraged to improve their data quality by implementing robust data governance protocols [21,23,31,41,42], adopting standardized data protocols to facilitate interoperability [24,34,35,39,45], and actively validating and verifying the accuracy of the data with clinicians and data scientists [30,41,54].

##### Strengthen Technological Infrastructures

The use of third-party hardware and software in AI solutions can limit control and raise security and privacy concerns [43]. Open-source software can improve transparency and accountability by allowing experts to identify vulnerabilities, but it can also make it easier for malicious actors to exploit them [35]. To mitigate this risk, hospitals can adopt validated open-source software with appropriate security and privacy measures, such as standardized databases and interoperability protocols [24,34,35,39,45].

#### Horizon 3: New Models of Care

The objective of horizon 3 is to restructure the clinical care model by harnessing the insights generated from AI analytics. While the main focus of this horizon is on clinicians and processes, fewer practical experiences are available for health organizations to help in shaping the implementation strategy.

Training end users to understand AI output is suggested to enhance the adoption of AI in hospitals (enabler 25; Table 3). Hospital leadership plays a pivotal role in facilitating the adoption of AI by providing strategic guidance, allocating necessary resources, and fostering a supportive environment for the implementation of AI initiatives (enabler 23; Table 3). Hospitals are suggested to appoint innovation managers to actively promote and facilitate the applications of AI, fostering uptake and driving the implementation process in health care (enabler 24; Table 3). Resourcing is the crucial enabler of AI integration, in particular adequate skill sets. Experienced clinicians who can interpret AI results are essential for ensuring that AI systems are used effectively and responsibly in health care organizations (enabler 22; Table 3). As a result, this can redefine the traditional models of care by advocating for evidence-based practices, patient-centered care, collaborative care, and continuous quality improvement to enhance patient outcomes and the overall quality of the care provided by health care organizations.

### Limitations

Our search strategy identified 26 studies that met the inclusion criteria. All 26 studies were conducted in high-income countries. As a result, the diversity and applicability of the findings to other health care systems were constrained.

By excluding regulatory frameworks from this review in the rapidly evolving regulatory landscape, we may have limited the important implementation guidelines that ensure patient safety and ethical use of AI provided by health care regulatory bodies.

We conducted a thorough examination of the reference lists in the included studies to ensure the inclusion of all relevant papers. Despite a valid research methodology, this approach may introduce publication bias, a factor to consider when appraising the study’s findings.

The methodological reporting of most studies included in this review was assessed as poor, potentially limiting the quality of the findings of this study. While consensus discussions were held after the quality assessment to mitigate potential discrepancies in the final evaluations, it is worth recognizing that this process is subjective and the perspectives of reviewers may evolve over time, resulting in variations when assessed by different individuals.

Although our intention was to identify successful implementations, it is possible that we missed significant enablers or barriers present in failed implementations.

### Conclusions

This review incorporated the identified enablers of and barriers to the implementation of AI into a 3-horizon framework to guide future implementations of hospital AI analytics to evolve practice toward an LHS. Successful AI implementation in hospitals requires a shift in conventional resource management to support a new AI implementation and maintenance strategy. Using analytics to enable better decisions in hospitals is critical to enable the ever-increasing need for health care to be met.

## Appendix

Multimedia Appendix 1 Enablers and barriers identified in previous reviews.

Multimedia Appendix 2 Search strategies across 4 databases for this review.

Multimedia Appendix 3 Quality assessment of the included studies.

Multimedia Appendix 4 Preferred Reporting Items for Systematic Reviews and Meta-Analyses (PRISMA) checklist.

## Abbreviations

AI: artificial intelligence
EMR: electronic medical record
LHS: learning health system
MeSH: Medical Subject Heading
MMAT: Mixed Methods Appraisal Tool
PRISMA: Preferred Reporting Items for Systematic Reviews and Meta-Analyses
RQ: research question

## Data Availability

All data generated or analyzed during this study are included in this published article (and its supplementary information files).

## References

[1] Williams F, Boren SA (2008). The role of the electronic medical record (EMR) in care delivery development in developing countries: a systematic review. Inform Prim Care.

[2] Mann DM, Chen J, Chunara R, Testa PA, Nov O (2020). COVID-19 transforms health care through telemedicine: evidence from the field. J Am Med Inform Assoc.

[3] Reeves JJ, Hollandsworth HM, Torriani FJ, Taplitz R, Abeles S, Tai-Seale M, Millen M, Clay BJ, Longhurst CA (2020). Rapid response to COVID-19: health informatics support for outbreak management in an academic health system. J Am Med Inform Assoc.

[4] Barlow J, Bayer S, Curry R (2006). Implementing complex innovations in fluid multi-stakeholder environments: experiences of ‘telecare’. Technovation.

[5] Sikka R, Morath JM, Leape L (2015). The Quadruple Aim: care, health, cost and meaning in work. BMJ Qual Saf.

[6] Kamel Rahimi A, Canfell OJ, Chan W, Sly B, Pole JD, Sullivan C, Shrapnel S (2022). Machine learning models for diabetes management in acute care using electronic medical records: a systematic review. Int J Med Inform.

[7] Yu KH, Beam AL, Kohane IS (2018). Artificial intelligence in healthcare. Nat Biomed Eng.

[8] Maddox TM, Rumsfeld JS, Payne PR (2019). Questions for artificial intelligence in health care. JAMA.

[9] Kelly CJ, Karthikesalingam A, Suleyman M, Corrado G, King D (2019). Key challenges for delivering clinical impact with artificial intelligence. BMC Med.

[10] Aung YY, Wong DC, Ting DS (2021). The promise of artificial intelligence: a review of the opportunities and challenges of artificial intelligence in healthcare. Br Med Bull.

[11] Yin J, Ngiam KY, Teo HH (2021). Role of artificial intelligence applications in real-life clinical practice: systematic review. J Med Internet Res.

[12] Nilsen P (2015). Making sense of implementation theories, models and frameworks. Implement Sci.

[13] He J, Baxter SL, Xu J, Xu J, Zhou X, Zhang K (2019). The practical implementation of artificial intelligence technologies in medicine. Nat Med.

[14] Guo Y, Hao Z, Zhao S, Gong J, Yang F (2020). Artificial intelligence in health care: bibliometric analysis. J Med Internet Res.

[15] Sullivan C, Staib A, McNeil K, Rosengren D, Johnson I (2020). Queensland Digital Health Clinical Charter: a clinical consensus statement on priorities for digital health in hospitals. Aust Health Rev.

[16] Etheredge LM (2007). A rapid-learning health system. Health Aff (Millwood).

[17] Mandl KD, Kohane IS, McFadden D, Weber GM, Natter M, Mandel J, Schneeweiss S, Weiler S, Klann JG, Bickel J, Adams WG, Ge Y, Zhou X, Perkins J, Marsolo K, Bernstam E, Showalter J, Quarshie A, Ofili E, Hripcsak G, Murphy SN (2014). Scalable collaborative infrastructure for a learning healthcare system (SCILHS): architecture. J Am Med Inform Assoc.

[18] Lim HC, Austin JA, van der Vegt AH, Rahimi AK, Canfell OJ, Mifsud J, Pole JD, Barras MA, Hodgson T, Shrapnel S, Sullivan CM (2022). Toward a learning health care system: a systematic review and evidence-based conceptual framework for implementation of clinical analytics in a digital hospital. Appl Clin Inform.

[19] Gama F, Tyskbo D, Nygren J, Barlow J, Reed J, Svedberg P (2022). Implementation frameworks for artificial intelligence translation into health care practice: scoping review. J Med Internet Res.

[20] Joshi M, Mecklai K, Rozenblum R, Samal L (2022). Implementation approaches and barriers for rule-based and machine learning-based sepsis risk prediction tools: a qualitative study. JAMIA Open.

[21] Sharma M, Savage C, Nair M, Larsson I, Svedberg P, Nygren JM (2022). Artificial intelligence applications in health care practice: scoping review. J Med Internet Res.

[22] Lee TC, Shah NU, Haack A, Baxter SL (2020). Clinical implementation of predictive models embedded within electronic health record systems: a systematic review. Informatics (MDPI).

[23] Wolff J, Pauling J, Keck A, Baumbach J (2021). Success factors of artificial intelligence implementation in healthcare. Front Digit Health.

[24] Chomutare T, Tejedor M, Svenning TO, Marco-Ruiz L, Tayefi M, Lind K, Godtliebsen F, Moen A, Ismail L, Makhlysheva A, Ngo PD (2022). Artificial intelligence implementation in healthcare: a theory-based scoping review of barriers and facilitators. Int J Environ Res Public Health.

[25] Platt JE, Raj M, Wienroth M (2020). An analysis of the learning health system in its first decade in practice: scoping review. J Med Internet Res.

[26] Page Matthew J, McKenzie Joanne E, Bossuyt Patrick M, Boutron Isabelle, Hoffmann Tammy C, Mulrow Cynthia D, Shamseer Larissa, Tetzlaff Jennifer M, Akl Elie A, Brennan Sue E, Chou Roger, Glanville Julie, Grimshaw Jeremy M, Hróbjartsson Asbjørn, Lalu Manoj M, Li Tianjing, Loder Elizabeth W, Mayo-Wilson Evan, McDonald Steve, McGuinness Luke A, Stewart Lesley A, Thomas James, Tricco Andrea C, Welch Vivian A, Whiting Penny, Moher David (2021). The PRISMA 2020 statement: an updated guideline for reporting systematic reviews. BMJ.

[27] Covidence systematic review software. Veritas Health Innovation.

[28] Hong QN, Gonzalez-Reyes A, Pluye P (2018). Improving the usefulness of a tool for appraising the quality of qualitative, quantitative and mixed methods studies, the Mixed Methods Appraisal Tool (MMAT). J Eval Clin Pract.

[29] Fervers B, Burgers JS, Voellinger R, Brouwers M, Browman GP, Graham ID, Harrison MB, Latreille J, Mlika-Cabane N, Paquet L, Zitzelsberger L, Burnand B (2011). Guideline adaptation: an approach to enhance efficiency in guideline development and improve utilisation. BMJ Qual Saf.

[30] Wilson A, Saeed H, Pringle C, Eleftheriou I, Bromiley PA, Brass A (2021). Artificial intelligence projects in healthcare: 10 practical tips for success in a clinical environment. BMJ Health Care Inform.

[31] Svedberg P, Reed J, Nilsen P, Barlow J, Macrae C, Nygren J (2022). Toward successful implementation of artificial intelligence in health care practice: protocol for a research program. JMIR Res Protoc.

[32] Subbaswamy A, Saria S (2020). From development to deployment: dataset shift, causality, and shift-stable models in health AI. Biostatistics.

[33] Pianykh OS, Langs G, Dewey M, Enzmann DR, Herold CJ, Schoenberg SO, Brink JA (2020). Continuous learning AI in radiology: implementation principles and early applications. Radiology.

[34] Leiner T, Bennink E, Mol CP, Kuijf HJ, Veldhuis WB (2021). Bringing AI to the clinic: blueprint for a vendor-neutral AI deployment infrastructure. Insights Imaging.

[35] Gruendner J, Schwachhofer T, Sippl P, Wolf N, Erpenbeck M, Gulden C, Kapsner LA, Zierk J, Mate S, Stürzl M, Croner R, Prokosch HU, Toddenroth D (2019). KETOS: clinical decision support and machine learning as a service - a training and deployment platform based on Docker, OMOP-CDM, and FHIR Web Services. PLoS One.

[36] Eche T, Schwartz LH, Mokrane FZ, Dercle L (2021). Toward generalizability in the deployment of artificial intelligence in radiology: role of computation stress testing to overcome underspecification. Radiol Artif Intell.

[37] Allen B, Dreyer K, Stibolt R Jr, Agarwal S, Coombs L, Treml C, Elkholy M, Brink L, Wald C (2021). Evaluation and real-world performance monitoring of artificial intelligence models in clinical practice: try it, buy it, check it. J Am Coll Radiol.

[38] Verma AA, Murray J, Greiner R, Cohen JP, Shojania KG, Ghassemi M, Straus SE, Pou-Prom C, Mamdani M (2021). Implementing machine learning in medicine. CMAJ.

[39] Wiggins WF, Magudia K, Schmidt TM, O'Connor SD, Carr CD, Kohli MD, Andriole KP (2021). Imaging AI in practice: a demonstration of future workflow using integration standards. Radiol Artif Intell.

[40] Wang B, Jin S, Yan Q, Xu H, Luo C, Wei L, Zhao W, Hou X, Ma W, Xu Z, Zheng Z, Sun W, Lan L, Zhang W, Mu X, Shi C, Wang Z, Lee J, Jin Z, Lin M, Jin H, Zhang L, Guo J, Zhao B, Ren Z, Wang S, Xu W, Wang X, Wang J, You Z, Dong J (2021). AI-assisted CT imaging analysis for COVID-19 screening: building and deploying a medical AI system. Appl Soft Comput.

[41] Strohm L, Hehakaya C, Ranschaert ER, Boon WP, Moors EH (2020). Implementation of artificial intelligence (AI) applications in radiology: hindering and facilitating factors. Eur Radiol.

[42] Soltan AA, Yang J, Pattanshetty R, Novak A, Yang Y, Rohanian O, Beer S, Soltan MA, Thickett DR, Fairhead R, Zhu T, Eyre DW, Clifton DA (2022). Real-world evaluation of rapid and laboratory-free COVID-19 triage for emergency care: external validation and pilot deployment of artificial intelligence driven screening. Lancet Digit Health.

[43] Sohn JH, Chillakuru YR, Lee S, Lee AY, Kelil T, Hess CP, Seo Y, Vu T, Joe BN (2020). An open-source, vender agnostic hardware and software pipeline for integration of artificial intelligence in radiology workflow. J Digit Imaging.

[44] Pierce JD, Rosipko B, Youngblood L, Gilkeson RC, Gupta A, Bittencourt LK (2021). Seamless integration of artificial intelligence into the clinical environment: our experience with a novel pneumothorax detection artificial intelligence algorithm. J Am Coll Radiol.

[45] Kanakaraj P, Ramadass K, Bao S, Basford M, Jones LM, Lee HH, Xu K, Schilling KG, Carr JJ, Terry JG, Huo Y, Sandler KL, Netwon AT, Landman BA (2022). Workflow integration of research AI tools into a hospital radiology rapid prototyping environment. J Digit Imaging.

[46] Jauk S, Kramer D, Großauer B, Rienmüller S, Avian A, Berghold A, Leodolter W, Schulz S (2020). Risk prediction of delirium in hospitalized patients using machine learning: an implementation and prospective evaluation study. J Am Med Inform Assoc.

[47] Davis SE, Greevy RA, Fonnesbeck C, Lasko TA, Walsh CG, Matheny ME (2019). A nonparametric updating method to correct clinical prediction model drift. J Am Med Inform Assoc.

[48] Blezek DJ, Olson-Williams L, Missert A, Korfiatis P (2021). AI integration in the clinical workflow. J Digit Imaging.

[49] Pantanowitz L, Quiroga-Garza GM, Bien L, Heled R, Laifenfeld D, Linhart C, Sandbank J, Albrecht Shach A, Shalev V, Vecsler M, Michelow P, Hazelhurst S, Dhir R (2020). An artificial intelligence algorithm for prostate cancer diagnosis in whole slide images of core needle biopsies: a blinded clinical validation and deployment study. Lancet Digit Health.

[50] Fujimori R, Liu K, Soeno S, Naraba H, Ogura K, Hara K, Sonoo T, Ogura T, Nakamura K, Goto T (2022). Acceptance, barriers, and facilitators to implementing artificial intelligence-based decision support systems in emergency departments: quantitative and qualitative evaluation. JMIR Form Res.

[51] Pou-Prom C, Murray J, Kuzulugil S, Mamdani M, Verma AA (2022). From compute to care: lessons learned from deploying an early warning system into clinical practice. Front Digit Health.

[52] Baxter SL, Bass JS, Sitapati AM (2020). Barriers to implementing an artificial intelligence model for unplanned readmissions. ACI Open.

[53] Sandhu S, Lin AL, Brajer N, Sperling J, Ratliff W, Bedoya AD, Balu S, O'Brien C, Sendak MP (2020). Integrating a machine learning system into clinical workflows: qualitative study. J Med Internet Res.

[54] Sendak MP, Ratliff W, Sarro D, Alderton E, Futoma J, Gao M, Nichols M, Revoir M, Yashar F, Miller C, Kester K, Sandhu S, Corey K, Brajer N, Tan C, Lin A, Brown T, Engelbosch S, Anstrom K, Elish MC, Heller K, Donohoe R, Theiling J, Poon E, Balu S, Bedoya A, O'Brien C (2020). Real-world integration of a sepsis deep learning technology into routine clinical care: implementation study. JMIR Med Inform.

[55] Wang F, Preininger A (2019). AI in health: state of the art, challenges, and future directions. Yearb Med Inform.

[56] Li JP, Liu H, Ting DS, Jeon S, Chan RV, Kim JE, Sim DA, Thomas PB, Lin H, Chen Y, Sakomoto T, Loewenstein A, Lam DS, Pasquale LR, Wong TY, Lam LA, Ting DS (2021). Digital technology, tele-medicine and artificial intelligence in ophthalmology: a global perspective. Prog Retin Eye Res.

[57] Sibbald M, Zwaan L, Yilmaz Y, Lal S (2024). Incorporating artificial intelligence in medical diagnosis: a case for an invisible and (un)disruptive approach. J Eval Clin Pract.

[58] Ehsani-Moghaddam B, Martin K, Queenan JA (2021). Data quality in healthcare: a report of practical experience with the Canadian Primary Care Sentinel Surveillance Network data. Health Inf Manag.

