# Supplementary material for: Implementing AI in Hospitals to Achieve a Learning Health System: Systematic Review of Current Enablers and Barriers
Source: J Med Internet Res. 2024 Aug 2;26:e49655. doi: 10.2196/49655 (PMC11329852; doi:10.2196/49655)
Supplement: Multimedia Appendix 1 [file jmir_v26i1e49655_app1.doc]

Enablers identified in the prior reviews

| Enablers of AI implementations in healthcare | | Meta study |
| --- | --- | --- |
| **People** | 1- Multidisciplinary team | Sharma et al(2020) |
|  | 2- Trained end user | Sharma et al(2020), Lee et al(2020), Chomutare et al(2022) |
|  | 3- Innovation champions | Sharma et al(2020), Lee et al(2020),Chomutare et al(2022) |
|  | 4- Hospital leadership | Chomutare et al(2022) |
| **Process** | 5- Co-design with end users | Sharma et al(2020) |
|  | 6- Staff training | Sharma et al(2020),Lee et al(2020),Chomutare et al(2022) |
|  | 7- Organisational resources | Sharma et al(2020),Lee et al(2020),Chomutare et al(2022) |
|  | 8- Seamless integration | Sharma et al(2020), Lee et al(2020) |
|  | 9- Robust performance monitoring and evaluation | Sharma et al(2020), Chomutare et al(2022) |
|  | 10- Provide incentives when using AI | Sharma et al(2020), Lee et al(2020) |
|  | 11- Limiting non-AI solutions | Sharma et al(2020) |
|  | 12- Evidence of clinical and economic AI-added value evidence | Sharma et al(2020),Wolff et al(2020), Chomutare et al(2022) |
|  | 13- Improved team communication | Sharma et al(2020) |
|  | 14- Addressing data shift | Lee et al(2020) |
| **Information** | 15- Data quality | Chomutare et al(2022) |
|  | 16- Data security | Lee et al(2020) |
|  | 17- Usability | Chomutare et al(2022) |
| **Technology** | 18- Continuous learning platform | Sharma et al(2020) |
|  | 19- Customisation capability | Sharma et al(2020), Lee et al(2020), Wolff et al(2020) |
|  | 20- Interoperability | Chomutare et al(2022) |

**Barriers identified in the prior reviews**

| Barries of AI implementations in healthcare | | Meta study |
| --- | --- | --- |
| **People** | 1- Inexperienced end-users with AI | Chomutare et al(2022) |
|  | 2- Lack of Clinicians’ trust | Lee et al(2020), Chomutare et al(2022) |
| **Process** | 3- Lack of standardised guidelines for AI implementation | Sharma et al(2020),Wolff et al(2020),Chomutare et al(2022) |
|  | 4- Inadequate continuous learning | Chomutare et al(2022) |
|  | 5- Insufficient performance assessment | Chomutare et al(2022) |
|  | 6- Complexity of maintenance | Chomutare et al(2022) |
|  | 7- Disruptive integration | Lee et al(2020),Chomutare et al(2022) |
|  | 8- Alert fatigue | Lee et al(2020),Chomutare et al(2022) |
|  | 9- Difficulties with understanding AI outputs | Chomutare et al(2022) |
|  | 10- Insufficient data pre-processing | Wolff et al(2020) |
| **Information** | 11- Poor data quality | Lee et al(2020),Wolff et al(2020),Chomutare et al(2022) |
|  | 12- Challenges with data availability | Lee et al(2020),Wolff et al(2020),Chomutare et al(2022) |
|  | 13- Data shift | Lee et al(2020) |
