# Supplementary material for: Implementing AI in Hospitals to Achieve a Learning Health System: Systematic Review of Current Enablers and Barriers
Source: J Med Internet Res. 2024 Aug 2;26:e49655. doi: 10.2196/49655 (PMC11329852; doi:10.2196/49655)
Supplement: Multimedia Appendix 2 [file jmir_v26i1e49655_app2.doc]

Search strategies across four databases for the present review

| **PubMed** | ("Artificial Intelligence"[Mesh:NoExp] OR "Machine Learning"[Mesh] OR "Neural Networks, Computer"[Mesh] OR AI[tiab] OR artificial intelligence[tiab] OR deep learning[tiab] OR machine learning[tiab] OR neural network*[tiab] OR “big data”) AND (Hospital OR “medical*” or “clinic*”or "Inpatient*" OR inpatient* OR in-hospital) AND (“Product lifecycle management”[ti] OR PLM[ti] OR Life-cycle[ti] OR “Life cycle”[ti] OR “smart manufacturing”[ti] OR Regulation[ti] OR regulatory[ti] OR “deploy*”[ti] OR “integrat*”[ti] OR “implement*”[ti] OR monitor[ti] OR drift [ti] OR post prediction[ti] ) |
| --- | --- |
| **Scopus** | ( TITLE ( "Product lifecycle management" OR plm OR life-cycle OR "Life cycle" OR "smart manufacturing" OR regulation OR regulatory OR "deploy*" OR "integrat*" OR "implement*" OR "monitor*" OR drift OR post prediction OR "Artificial Intelligence" OR "Artificial Intelligence" OR "AI") ) AND ( TITLE-ABS ( "hospital*" OR "medical*" OR "clinic*" OR healthcare OR health) ) AND ( ( TITLE-ABS ( "Artificial Intelligence" OR "AI" OR "Machine Learning" OR "Neural Networks, Computer" OR "Neural language*" OR "artificial intelligence" OR "deep learning" OR "machine learning" OR "neural network*" OR "big data") ) ) |
| **Web of Science** | ((TI=( "Product lifecycle management" OR plm OR life-cycle OR "Life cycle" OR "smart manufacturing" OR regulation OR regulatory OR "deploy*" OR "integrat*")) AND TS=(hospital OR “medical*” OR “clinic*” OR healthcare OR health OR monitor Or drift OR post prediction)) AND (TS=("Artificial Intelligence" OR "Machine Learning" OR "Neural Networks, Computer" OR "deep learning" OR "machine learning" OR "neural network*" OR “big data”) OR TI=(AI)) |
| **IEEE** | ("Document Title":"Product lifecycle management" OR "Document Title": plm OR "Document Title": life-cycle OR "Document Title": "Life cycle" OR "Document Title": "smart manufacturing" OR "Document Title": regulation OR "Document Title": regulatory OR "Document Title": deployment OR "Document Title": integration) AND ("All Metadata":hospital OR "All Metadata": medical OR "All Metadata": clinical) AND ("All Metadata":"Artificial Intelligence" OR "All Metadata": "Machine Learning" OR "All Metadata": "Neural Networks, Computer" OR "All Metadata": "artificial intelligence" OR "All Metadata": "deep learning" OR "All Metadata": "machine learning" OR "All Metadata": "neural network*" OR "All Metadata": "big data" OR "All Metadata": monitor OR "All Metadata": drift OR "All Metadata":"post prediction") |
